# Serum and follicular fluid levels of soluble receptor for advanced glycation end-products in women with and without polycystic ovary syndrome

**DOI:** 10.1186/s13048-023-01224-z

**Published:** 2023-06-30

**Authors:** Neda Emami, AliReza Alizadeh, Arezoo Maleki-Hajiagha, Alireza Dizavi, Samira Vesali, Ashraf Moini

**Affiliations:** 1 Department of Gynecology and Obstetrics, Tehran University of Medical Sciences, Tehran, Iran; 2 Department of Embryology, Reproductive Biomedicine Research Center, Royan Institute for Reproductive Biomedicine, ACECR, Tehran, Iran; 3 gyn-medicum, Center for Reproductive Medicine, Göttingen, Germany; 4 Institute of Pharmacology and Toxicology, University Medical Center Göttingen, Göttingen, Germany; 5 Department of Anatomy, School of Medicine, Tehran University of Medical Scienes, Tehran, Iran; 6 Department of Clinical Nutrition and Dietetics, Faculty of Nutrition and Food Technology, National Nutrition and Food Technology, Research Institute Shahid Beheshti University of Medical Sciences, Tehran, Iran; 7 Reproductive Epidemiology Research Center, Royan Institute for Reproductive Biomedicine, ACECR, Tehran, Iran; 8 Department of Endocrinology and Female Infertility, Reproductive Biomedicine Research Center, Royan Institute for Reproductive Biomedicine, ACECR, Tehran, Iran; 9 Breast Disease Research Center (BDRC), Tehran University of Medical Sciences, Tehran, Iran; 10 Department of Gynecology and Obstetrics, Arash Women’s Hospital, Tehran University of Medical Sciences, P.O Box: 1653915981, Tehran, Iran

**Keywords:** Extracellular Fluid, IVF, Receptor for Advanced Glycation End Product, Polycystic Ovary Syndrome

## Abstract

**Background:**

Advanced glycation end products (AGEs) are known to associate with the pathogenesis of several chronic diseases via interaction with their corresponding receptor (RAGE). The soluble forms of RAGE (sRAGE) are considered as anti-inflammatory agents by inhibiting the consequent adverse effects of AGE. We aimed at comparing sRAGE levels in the follicular fluid (FF) and serum of women with or without Polycystic Ovary Syndrome (PCOS) who underwent controlled ovarian stimulation for in vitro fertilisation (IVF).

**Methods:**

A total of forty-five eligible women (26 non-PCOS (control) and 19 patients with PCOS (case)) were included the study. sRAGEs in FF and blood serum were measured using ELISA kit.

**Results:**

No statistically significant differences were found in FF and serum sRAGE between case and control groups. Correlation analysis showed a significant and positive relationship between serum levels of sRAGE and FF sRAGE in PCOS (*r* = 0.639; *p* = 0.004), in control participants (*r* = 0.481; *p* = 0.017), and in total participants (*r* = 0.552; *p* = 0.000). Data revealed a statistically significant difference in FF sRAGE concentration among all participants by body mass index (BMI) categories (*p* = 0.01) and in controls (*p* = 0.022). Significant differences were found for all the nutrients and AGEs consumption according to Food Frequency Questionnaire in both groups (*p* = 0.0001). A significant reverse relationship was found between FF levels of sRAGE and AGE in PCOS (*r* = -0.513; *p* = 0.025). The concentration of sRAGE in serum and FF is the same in PCOS and control.

**Conclusion:**

The present study revealed for the first time that there are no statistically significant differences between the concentration of serum sRAGE and FF sRAGE among Iranian women with and without PCOS. However, BMI and dietary intake of AGEs have more significant effects on sRAGE concentration in Iranian women. Future studies in developed and developing countries with larger sample sizes are required to determine the long-term consequences of chronic AGE over consumption and the optimal strategies for minimizing AGE-related pathology, specifically in low income and developing countries.

## Introduction

As an endocrine disorder, Polycystic ovary syndrome (PCOS) is the most common cause of anovulatory infertility. It has been reported that more than 15% of reproductive-aged women are suffering from PCOS [1, 2]. PCOS diagnosis is based on hyperandrogenism, oligo/anovulation, and polycystic ovaries morphology in ultrasound assessment. Despite the poorly defined aetiology of PCOS, obesity, sedentary life style, and unhealthy nutritional pattern are known to be key role players. One of the most important environmental parameter discovered to be potentially associated with both reproductive and metabolic dysfunctions of PCOS, is the dietary intake of advanced glycation end products (AGEs) [3, 4]. Other than being endogenously formed, AGEs are found abundantly in fast-food meals and products [5]. Also, AGEs are produced following non-enzymatic modifications from proteins, nucleic acids, or lipids, they are basically and extremely pro-inflammatory [6, 7]. As mentioned, the glycoxidation reaction, also called browning or Maillard reaction [8], leads to elevated serum AGEs levels and is associated with various pathologies like atherosclerosis, aging, and rheumatoid arthritis to endocrinopathies such as insulin resistance, hyperglycaemia, and diabetes. It was pointed out that PCOS has also been detected to be closely linked to high levels of AGEs [7].

Upon deposition in various tissues and organs, such high levels of circulating AGEs lead to cellular damages [9]. The putative mechanism of action for AGEs starts by binding to the extracellular matrix (receptor independent) or to receptor for advanced glycation end products (RAGE) (receptor mediated) [10]. Binding of AGEs with its receptor, activates the downstream inflammatory and apoptotic signalling pathways [11]. Studies have reported raised AGEs’ levels and consequently increased expression of their corresponding pro-inflammatory receptors, RAGEs, in the ovarian tissue of women with PCOS [10]. Supporting clinical data also shows decreased serum AGE levels following orlistat treatmant in POCS patients [12]. A high-fat diet has also been demonstrated to increase the circulating and visceral fat AGEs in rats [13]. Similarly, food containing low amounts of AGE could lower the risk of hormonal and metabolic abnormalities and also ameliorate levels of oxidative stress biomarkers in patients with POCS [14].

The soluble form of AGE receptor, sRAGE, is secreted to the extracellular matrix and is further detected in blood and follicular fluid (FF) [15]. Binding of soluble forms of the receptor the circulating AGEs, would prevent their further attachment to RAGEs. Hence, sRAGEs are considered as anti-inflammatory agents as they inhibit the adverse effects of AGE-RAGE pro-inflammatory signalling in the target cells [16]. Similarly, the higher level of the sRAGE, the more anti-inflammatory effects against AGEs are expected [17]. Interestingly, the serum and FF levels of sRAGE have been reported lower in women suffering from PCOS which were inversely associated with their body mass index (BMI) and insulin resistance as well [18].

High sRAGE could improve IVF outcomes, as decreases the required doses of gonadotropins for controlled ovarian stimulation (COS) and increases the number of high quality mature retrieved oocytes and pregnancy rates [16]. Obesity is the well-known risk factor of various health issues and probably one of the most accurate predictors of reproductive and metabolic dysfunction in PCOS alongside with AGEs. It is also inversely associated with response to treatment in PCOS patients. Even regardless of reproductive complications of PCOS, the association of obesity and AGEs with long term metabolic and cardiovascular complications of PCOS should not be simply neglected. Thus, changes in dietary patterns and healthy weight loss are considered the first line treatment options for management of PCOS [19]. The current case–control study aimed at comparing sRAGE levels in the FF and serum of Iranian infertile women with or without PCOS underwent IVF cycle considering age and BMI.

## Method and materials

### Study design and population

The study was approved by the ethics committee of Tehran university of Medical Science (IR.TUMS.MEDICINE.REC.1401.066). The study participants were recruited among reproductive aged women underwent COS followed by oocyte retrieval for IVF at Erfan and Arash Hospitals, Tehran, Iran, between June 2022 and September 2022. A total of 45 participants were considered to be eligible, including 26 women with a diagnosis of male factor infertility as the control group (non-PCOS) and 19 women with PCOS based on 2003 Rotterdam criteria [20], as the case group. PCOS diagnosis was reporetd by the medical practitioners affiliated to Tehran university of Medical Science, Tehran, Iran. The exclusion criteria included history of diabetes mellitus, chronic kidney disease, any type of chronic metabolic diseases, endometriosis, consumption of any medication affecting glucose and lipid metabolism, alcoholism, and smoking.

### COS Protocol and Oocytes retrieval

Pituitary desensitization was performed according to similar standards at both hospitals in both groups, using gonadotropin-releasing hormone (GnRH) antagonists. The COS protocols were flexible and follicular growth was stimulated by subcutaneous injection of gonadotropins (GONAL-F 150 IU/d, Merck, Germany) through 3–5 day of mensuration. The size of follicles was monitored with transvaginal ultrasounds after 5–7 day. When at least two mature follicles with a mean diameter of 15- 17 mm, were detected in ultrasound, final follicular maturation were triggered by injection of 10,000 IU recombinant human chorionic gonadotropin (hCG) (OVITRELLE 6500 IU/d, Merck, Germany). Thirty-four hours later, transvaginal ultrasound-guided oocyte retrieval was performed and FF was collected from the fluid obtained from the first large aspirated follicle that contained no blood. The FF was further centrifuged at 15,000 g for 5 min and the supernatant was immediately frozen at –80° C.

For assessing serum sRAGE, a sample of peripheral blood was collected in a tube with no anticoagulants. Afterwards, the naturally formed clot in the room temperature after 15–30 min was removed by centrifuging at 2000 g for 10 min in a refrigerated (4 °C) centrifuge. The resulting serum supernatant was also stored at -80 °C.

### Study variables and measurements

Demographic and anthropometric characteristics (age, weight, height,...) were recorded. Also, the food frequency was recorded by a trained researcher in special questionnaires. A validated 168-item food frequency questionnaire (FFQ) was filled by participants on the first examination to obtain information on their usual and routine diet over the last year [21]. An experienced nutritionist was assigned to extract and record nutritional information through questionnaire. The items of question included the intake frequency for each food during the past year on a daily, weekly, or monthly basis. Portion sizes was also inquired and recorded by conversion to daily grams. Energy and other nutrient contents were calculated using the USDA food composition tables (FCT) [22], dietary energy, macronutrients, micronutrients, and AGEs contents. Dietary AGEs scores were calculated using AGEs content in 91 food items of 168-FFQ based on the studied conducted by Uribarry et al. in 2010 [23]. Uribarry et al.’s study and previous studies had provided complete information about AGEs content of 546 food items and also in the same food item with various cooking methods. For some traditional foods, the similar items of food in that study were chosen. Finally, the AGEs value of all food items was summed for every person and the values were reported.

Serum and FF sRAGEs were measured using commercially available ELISA kits, ZELLBIO ZB-10027C-H9648 (GmbH) (ZELLBIO, Hamburg, Germany). The intra-and inter-assay CV were < 10% and < 12%, respectively.

### Statistical analysis

The mean and standard deviations (S.D) were used to describe variables that were all normally distributed. Student’s t-test and one-way ANOVA test were applied for comparisons between the PCOS group and the control group, and for comparing serum sRAGE,sRAGE FF, and AGE by BMI and age categories, respectively. Pearson correlation analysis was used for relationship between serum sRAGE and sRAGE FF, AGE, and clinical parameters such as age and BMI. All statistical analyses were performed using SPSS software (version 12.0 for Windows; SPSS Inc.). We considered p < 0.05 to indicate statistical significance.

## Results

The demographics and clinical data from all 45 participants who completed the study are presented in Table 1. The study population was comprised of 19 PCOS cases (42.2%) and 26 non-PCOS (control) subjects (57.8%). The mean (SD) age and BMI of participants were calculated 34.02 (7.03) years and 25.99 (3.98) kg/m^2^, respectively. Statistically significant differences were observed between both groups according to age and BMI. The mean (SD) age of PCOS participants was 29.32 (5.54) years and lower than controls [37.46 (5.97)] (*p* = 0.000). PCOS women showed higher BMI [27.39 (4.40)] than controls [24.98 (3.38) kg/m2] (*p* = 0.045). As shown in Table 1, total mean (SD) scores of serum sRAGE and sRAGE FF were 2.89 (1.37) and 3.53 (1.16) ng/mL, respectively. No statistically significant differences were observed in mean (SD) concentration of serum sRAGE and sRAGE FF between two groups. Total mean (SD) scores of AGE was 10499.77 ± 6949.69 kU/100gr. Mean comparison of AGE between women with PCOS and controls was statistically significant. The mean±SD of AGE in PCOS was 7490.68 ± 3782.91 and in controls 12698.71 ± 7141.41 kU/100gr (*p* = 0.006).

**Table 1:** Demographic and clinical characteristics of the patients and serum/ follicular fluid (FF) sRAGE in the case (PCOS) and control (non-PCOS) groups

	PCOS *N* = 19	Non-PCOS *N* = 26	Total	*P*-value*
Age (year)	**29.3** (5.54)	**37.4** (5.97)	34.02 (7.03)	**0.000**
Body mass index (kg/m^2^)	**27.3** (4.40)	**24.9** (3.38)	25.99 (3.98)	**0.045**
Serum sRAGE (ng/mL)	2.83 (1.45)	2.93 (1.33)	2.89 (1.37)	0.814
FF sRAGE (ng/mL)	3.56 (1.33)	3.51 (1.04)	3.53 (1.16)	0.891
***AGE*** (kU/100gr)	**7490** (3782.91)	**12,698** (7141.41)	10,499 (6449.69)	**0.006**

Values are presented as the mean (SD). The PCOS group compared with the control. *Significance at *P* < 0.05; Means in bold were significant

The nutrient intakes determined by the FFQ were expressed using mean and standard deviation and reported in Table 2. The significance of the differences of dietary intake was assessed by Student’s t-test is also reported in the table. Significant differences were found for all the nutrients between case and control groups (Table 2; *p* = 0.0001).

**Table 2:** The nutrient intake comparison between two groups by the food frequency questionnaire (case: PCOS; *N* = 19 and control: non-PCOS; *N* = 26)

	Group	Mean	Std. Deviation	*p*-value
Energy	PCOS	1785.9683	1050.68336	0.0001
	Non- PCOS	2247.4709	970.56123	
Protein	PCOS	116.5873	44.21918	0.0001
	Non- PCOS	134.9054	37.70017	
Carbohydrate	PCOS	258.2496	162.72037	0.0001
	Non- PCOS	300.9805	153.24338	
FAT	PCOS	50.8517	34.23060	0.0001
	Non- PCOS	80.8391	40.09641	
Cholesterol	PCOS	413.8233	84.85447	0.0001
	Non- PCOS	558.8543	168.11670	
Saturated FA^a^	PCOS	15.0141	10.13449	0.0001
	Non- PCOS	25.8195	13.01400	
MUFA	PCOS	36.0351	14.03185	0.0001
	Non- PCOS	48.1504	12.46719	
PUFA	PCOS	11.0818	7.12683	0.0001
	Non- PCOS	15.1773	8.79612	
oleic	PCOS	16.7719	12.86984	0.0001
	Non- PCOS	24.9040	11.60470	
linoleic	PCOS	9.0591	6.89240	0.0001
	Non- PCOS	12.4790	7.84236	
linolenic	PCOS	2.0907	.69045	0.0001
	Non- PCOS	2.9107	.93640	
EPA	PCOS	.0502	.12160	0.0001
	Non- PCOS	.0269	.02704	
DHA	PCOS	2.9625	1.31632	0.0001
	Non- PCOS	3.4206	.08447	
Trans fatty acids	PCOS	.0000	.00000	0.0001
	Non- PCOS	.0011	.00316	

^a^*FA* Fatty acids, *MUFA* Mono Unsaturated Fatty Acid, *PUFA* Poly Unsaturated Fatty Acid, *EPA* Eicosapentaenoic Acid, *DHA* Docosapentaenoic Acid

Correlation analysis showed a significant and positive association between serum sRAGE and FF sRAGE in case group (*r* = 0.639; *p* = 0.004), in control group (*r* = 0.481; *p* = 0.017), and in total participants (*r* = 0.552; *p* = 0.000). Among all participants, we found significant correlations between FF sRAGE and serum sRAGE (*r* = 0.552; *p* = 0.000), FF sRAGE and age (*r* = -0.346; *p* = 0.023), and finally, FF sRAGE and BMI (*r* = 0.361; *p* = 0.017). Among all cases in PCOS group, a significant relationship was observed between FF sRAGE and age (*r* = -0.532; *p* = 0.019), and between FF sRAGE and BMI (*r* = 0.463; *p* = 0.046) (Table 3).

**Table 3:** Correlation between serum levels of sRAGE and FF sRAGE, and demographic and clinical characteristics of the patients in the PCOS and control groups

	*Age (year)*		*Body mass index (kg/m*^*2*^*)*	
	*r*	p-value*	*r*	p-value*
*PCOS*				
*Serum sRAGE (ng/mL)*	0.093	0.714	0.318	0.198
FF sRAGE *(ng/mL)*	**-0.532**	0.019*	**0.463**	0.046*
*Non-PCOS*				
*Serum sRAGE (ng/mL)*	0.255	0.217	0.209	0.306
FF sRAGE *(ng/mL)*	0.255	0.228	-0.296	0.160
*Total*				
*Serum sRAGE (ng/mL)*	0.171	0.268	0.241	0.116
FF sRAGE *(ng/mL)*	**-0.346**	0.023*	**0.361**	0.017*

Statistical significance using the Pearson correlation coefficient. *Significance at *P* < 0.05, *r* in bold were significant

Correlation analysis showed a significant and positive relationship between AGE and age in PCOS group (*r* = 0.944; *p* = 0.000), in non-PCOS participants (*r* = 0.923; *p* = 0.000), and in total participants (*r* = 0.906; *p* = 0.000) (Table 4). Among all the PCOS, there was a significant reverse relationship between follicular fluid levels of sRAGE and AGE (*r* = –0.513; *p* = 0.025) (Table 4).

**Table 4:** Correlation between AGE and demographic and clinical characteristics of the patients and follicular fluid and serum levels of sRAGE in the PCOS and control groups

	***Age (year)***	***Body mass index (kg/m***^***2***^***)***	***Serum sRAGE (ng/mL)***	**FF sRAGE *(ng/mL)***
***PCOS***				
*r*	**0.944**	-0.277	0.017	**-0.513**
*p-value*	0.000*	0.250	0.946	0.025*
***Non-PCOS***				
*r*	**0.923**	0.102	0.163	-0.239
*p-value*	0.000*	0.619	0.426	0.260
***Total***				
*r*	**0.906**	-0.140	0.120	-0.299
*p-value*	0.000*	0.357	0.436	0.052

Statistical significance using the Pearson correlation coefficient. *Significance at *P* < 0.05, *r* in bold were significant

**Fig. 1:**
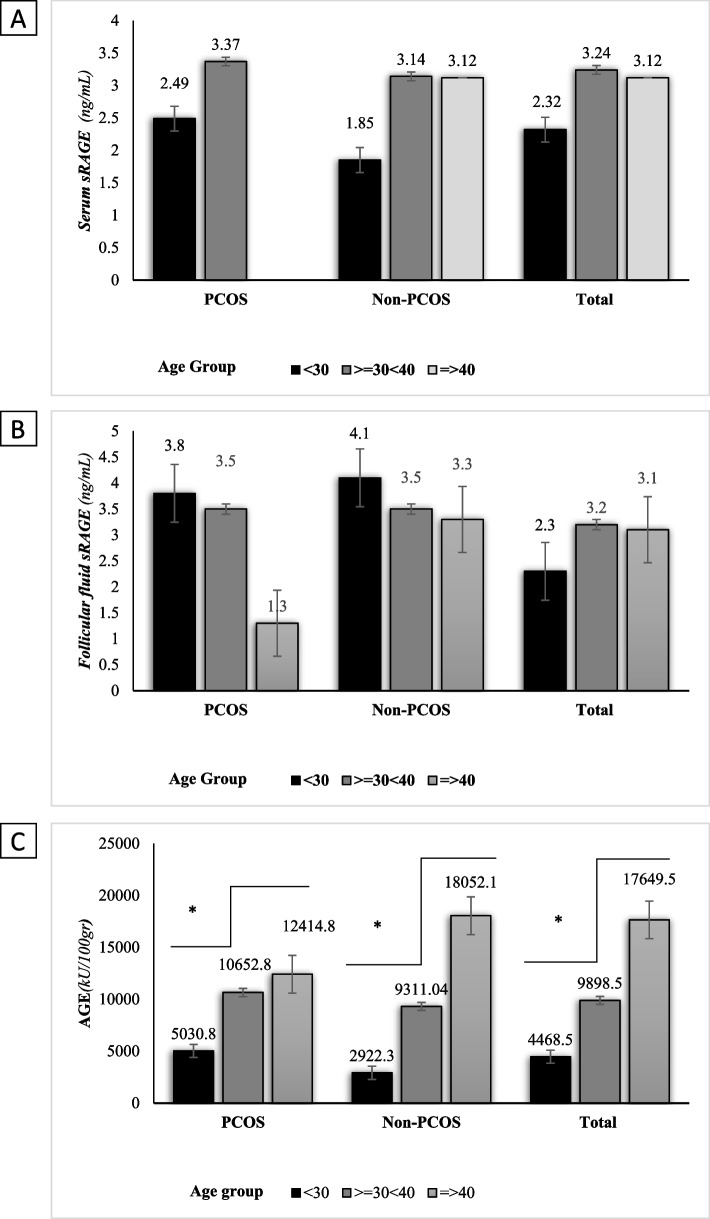
Comparison of serum levels of sRAGE (**A**), follicular fluid sRAGE (**B**), and AGE (**C**) between PCOS and non-PCOS groups by age groups. Comparisons were made by ANOVA

Upon adjusting serum sRAGE and sRAGE FF by age (< 30, >  = 30 < 40, =  > 40 years) and BMI categories (< 25, >  = 25 kg/m^2^), it was found that among all participants, there was no statistically significant differences in mean (SD) of serum (*p* = 0.131) and FF (*p* = 0.270) sRAGE by age groups. Similar to the total population, in women with and without PCOS separately, there was no statistically significant differences in mean (SD) of serum sRAGE (PCOS: *p* = 0.218, controls: *p* = 0.216) and FF sRAGE (PCOS: *p* = 0.191, controls: 0.491) by age groups (Fig. 1a, b).

**Fig. 2:**
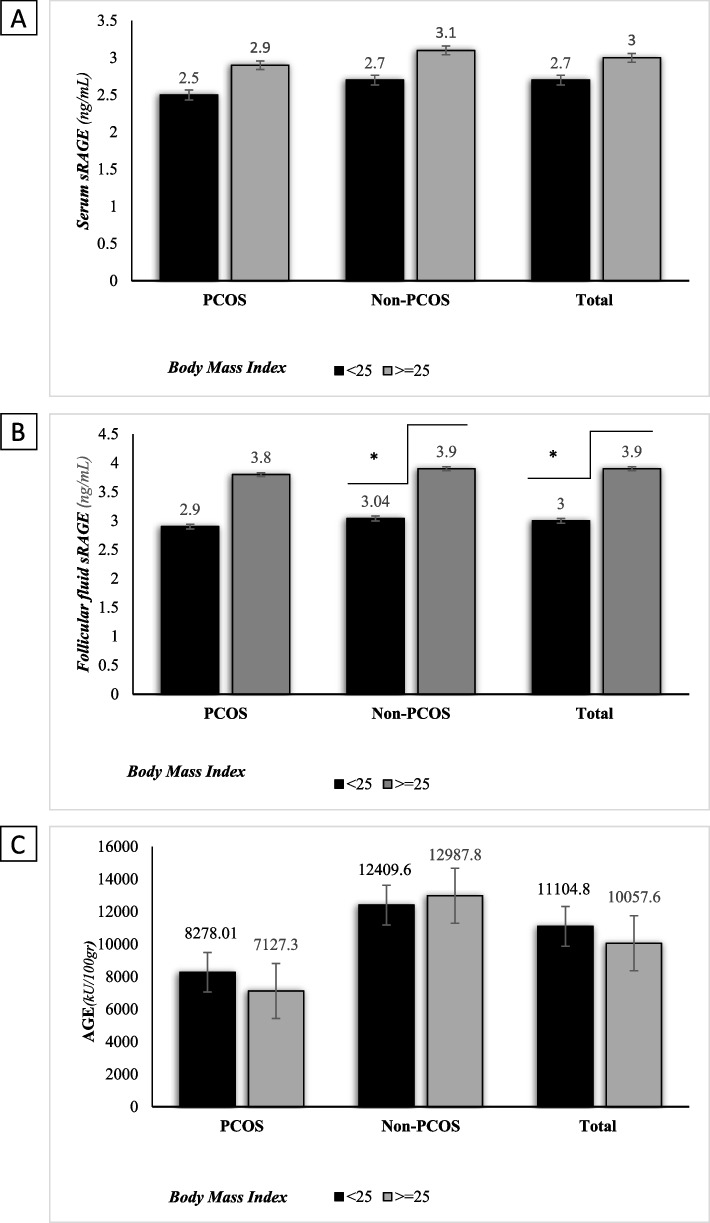
Comparison of serum levels of sRAGE (**A**), follicular fluid sRAGE (**B**), and AGE (**C**) between PCOS and non-PCOS groups by BMI categories. Comparisons were made by t-test

Our data also revealed a statistically significant difference in FF sRAGE concentrations among total population by BMI categories (*p* = 0.01). Similar to total population, a statistically significant difference was found in FF sRAGE concentration by BMI categories in control group (*p* = 0.022) (Fig. 2b).

Upon adjustment of AGE by age (< 30, >  = 30 < 40, =  > 40 years) and BMI categories (< 25, >  = 25 kg/m2), it was found that among all the participants, there was statistically significant differences in mean (SD) comparison of AGE by age groups (*p* = 0.000). Similar to total population, in women with and without PCOS, there was statistically significant differences in mean (SD) comparison of AGE (*p* = 0.000 and *p* = 0.000, respectively) by age groups (Fig. 1c). Data revealed that there was no statistically significant difference in AGE concentration among total population by BMI categories (*p* = 0.596). Similar to total population, in women with and without PCOS, there was no statistically significant difference in AGE concentration by BMI categories (*p* = 0.553 and *p* = 0.841, respectively) (Fig. 2c).

## Discussion

There is a growing body of evidence supporting negative effects of AGE–RAGE axis on folliculogenesis and follicular microenvironment and consequently ovarian dysfunction and poor IVF outcomes. However the mechanisms behind this association are still unclear [3, 24]. The present study found no differences in FF or serum sRAGE between the case and control groups, but it revealed the statistically significant difference between FF sRAGE concentration in total population by BMI (> 25) categories. Also, it seems that BMI and dietary intake of AGEs have more significant effect on dramatic changes of sRAGE than the foods quantity in women with and without PCOS.

It has been suggested that among all effective items, the most important determinant and most independent predictive of sRAGE levels in adolescents is BMI. Similarly, it was shown in a cross-sectional study by He et al. that there was reported a positive correlatiuon between sRAGE, obesity, and metabolic syndrome (MetS) in Taiwan adolescents. They found that plasma sRAGE level is associated with obesity and MetS [25].

Our results are in accordance with the findings of Lorenzi et al. who showed that sRAGE and anti-sRAGE levels are increased in morbid obesity (mean BMI = 46) in a population composing of men and women. Interestingly, they observed that a weight loss following gastric bypass led to a decrease in the levels of both evaluated factors [26]. In contrast, Merhi et al. pointed out a negative association between serum sRAGE concentrations and BMI [15]. Wang et al. in 2016, also showed that in women with PCOS (mean BMI = 24) a significantly lower FF sRAGE levels compared to control (BMI = 25) as well as all participants [27]. The higher levels of sRAGE in our obese subjects contradicted the results of the study by Brix et al. on 85 subjects comprising of 75 females and 10 males at the BMI of 41 and 45, respectively, individuals with morbid obesity after bariatric surgery and lost wight, showed decreased in sRAGE [28]. Horwitz et al**.** showed in 2016 the same result as Brix et al.’s study [29]. Although the role of soluble RAGE as a biomarker investigated in previous studies, it was demonstrated no change in the serum sRAGE level after neither gastric surgery not 6 months post operation [30]. Similarly, Hagen et al. in 2015, despite putting the subjects on a low-calorie diet which led to decreased BMIs, the sRAGE remained unchanged [31]. Regarding such controversial findings, we assume that the contradictions probably root in the inconsistent inclusion/exclusion criteria in the numerous studies on sRAGE. So, it seems necessary to focus on other less-studied characteristics in reproductive-aged women. Indeed, controversial issue needs further investigations on the association between BMI and sRAGE levels.

Although the exact role of sRAGE in the FF is still unclear, it could probably be linked to the AGE-RAGE axis in follicles as certain RAGE polymorphisms have been demonstrated to be linked to higher sRAGE levels [32]. This finding suggests that sRAGE is genetically regulated and therefore, could be used as not only a PCOS marker but also a potential therapeutic target. Also, there are other factors potentially affecting the level of sRAGE including age, RAGE polymorphisms, smoking, BMI, and ethnicity [17, 33].

It is surprising to note that BMI > 25 in either control or total population was directly related to an increased sRAGE in FF but not in serum. Uniquely, FF is a more sensitive than serum in relation to demographic characteristics and sRAGE concentration in our study. Although numerous studies have shown the effects of various foods on the concentration of sRAGE in serum and FF to be quite significant, we found no alteration in the concentration of sRAGE in case and control groups despite the significant difference in their amount of food consumption. Similarly, Irani and Mehri in a systematic review pointed out that vitamin D3 has a protective effect in women with PCOS by inhibiting the inflammatory signalling cascade through AGE through increasing circulating sRAGE [34]. Garg et al. also showed an increased level of serum sRAGE after 8-week supplemented with vitamin D3 in vitamin D deficient women with or without PCOS [35].

The changes in sRAGE concentration exerted by food intake could be explained by a reduced AGE substrate availability, taking food rich in anti-AGE compounds (antioxidant and food of antiglycation properties), increased sRAGE concentration, and changes in cooking methods [36]. Regarding the nutritional differences between the two groups in the current study, the significant difference was the higher consumption of carbohydrates in Iranian non-PCOS women which is quite thought-provoking. The possibility of a similar association between diet and risk for PCOS has not been examined widely. Previously, Douglas et al. showed PCOS women compared with matched control women, exhibited a dietary pattern that was marked by consumption of a greater number of specific foods with a high glycemic index [37].

Little information exists on the AGEs levels for women suffering from PCOS. According to recent study in 2017, PCOS patient, compared to controls of matched age, has a higher intake of fat and carbohydrates and lower intake of protein, fibre and polyunsaturated fatty acids compared to control group [38]. The PCOS women in the current study have less food consumption than the control group; because they have been treated for a long time as well as younger than control group.

In Carmina et al.’s study, women with PCOS from United States and Italy were compared based on their compared energy and macronutrient intake. Interestingly, their results showed a significantly higher saturated fat intake in United States’ patients compared to the Italian group [39]. Based on the literature, Iranians have been shown to get most of their daily energy from carbohydrate consumption and the total daily energy intake derived from carbohydrates in Iranian population is higher than 60% compared to 50% reported in western diet [40]. Altogether, it seems that besides factors like age, BMI and genetics, availability of substrates for AGE production can greatly affect the concentration of sRAGE while the quantity of food consumption had no effect in either PCOS or non-PCOS women.

Although no correlation was found between age and sRAGE in the present study, it seems that this relationship can start to change at certain ages, as Prakash et al. showed a decline in sRAGE level by age which was inversely associated with BMI and fat free mass (FFM) in healthy subjects [41]. An interesting study conducted on healthy centenarians demonstrated a higher plasma sRAGE level compared to healthy young individuals. This can support the idea of sRAGE being a marker of healthy aging and longevity. It would also be helpful to specify the contribution of each factor to sRAGE decline and the possible association to age-related disease risk factors [42].

Our experiment has a few constraints. Small sample size was the restrictions of the current study for women with PCOS. Additionally, we focused on Iranian women with PCOS. It would have been interesting to compare AGEs and sRAGE across IVF as well as further studies with larger sample sizes in other countries which could confirm these results in women with PCOS alongside measured several hormone and vitamin D levels.

## Conclusion

The present study revealed for the first time that there are no statistically significant differences between the concentration of serum sRAGE and FF sRAGE among Iranian women with and without PCOS. However, BMI and dietary intake of AGEs have more significant effects on sRAGE concentration in Iranian women. Future studies in developed and developing countries with larger sample sizes are required to determine the long-term consequences of chronic AGE over consumption and the optimal strategies for minimizing AGE-related pathology, specifically in low income and developing countries.

## Data Availability

The datasets used and/or analyzed during the current study are available from the corresponding author on reasonable request.
